# 

**Published:** 1921-04-09

**Authors:** 


Mrs. Mariabella Fry, of Failand, Somerset, has bequeathed £2,000 to the Clevedon Convalescent Homes to endow two beds; £1,500 to the Bristol General Hospital to endow a maternity bed for married mothers; £1,000 each to the Bristol Royal Hospital for Sick Children and Women, the Weston-super-Mare Children's Convalescent Home, the Bristol Private Hospital for Women and Children, and to the National Union of Trained Nurses in trust for the Somerset and Bristol area.

### The Almoner's Work at King's.

An interesting article on the almoner's record at King's College Hospital was contributed by a correspondent to a recent issue of the *South London Press*. The writer, enlarging on the growth of this branch of hospital work, remarked that the almoner's staff at King's consisted of four trained almoners, a maternity social worker, four clerks, and four students working in the department, which is "recognised as a training-school under the Hospital Almoners' Council." Beginning, to prevent hospital abuse, seventeen years ago, an assistant almoner was appointed six years later. Now every fresh patient, "except those attending some of the special departments for which as yet no member of the almoner's staff is available," is interviewed, and referred, after report to the doctor, to a panel practitioner, to the Poor Law, to district nurses, if admission to the hospital must be postponed, to local relief committees, to the Guardians, the Tuberculosis Care Committee, the Juvenile Advisory Committee (refractory boys and girls), or the War Pensions Committee, as the case requires. The in-patients are visited daily in the wards, and if a patient is worrying, say, because his place will not be kept open for him by his employer, the almoner does what she can to apprise the employer of the facts and to this extent to remove the anxiety. It is the mark of the great development which has taken place that the almoner's work now concerns in-patients and out-patients in equal, though different, ways.

### Parliamentary Medical Committee.

The Medical Committee of the House of Commons, of which Capt. Walter Elliot is secretary, met on March 21, 1921, in Room 13, House of Commons, Dr. Nathan Raw in the chair.

Sir Philip Magnus submitted correspondence with the Ministry of Health on the subject of security of tenure of medical officers of health. A Bill drafted some years ago (Public Health Officers' Bill) was considered. Sir Philip Magnus agreed to have the Bill brought up to date and submitted to the Committee at a subsequent meeting.

A deputation from the Medico-Political Union (Dr. Welply, Dr. Gordon Ward, Dr. Macdonald, and Dr. Greig) was then heard. Dr. Gordon Ward submitted objections to the new medical record cards. Dr. Greig submitted objections to the new regulations of the Ministry of Health, to come into force next year, concerning the transfer of practices. A discussion then took place lasting about an hour. Dr. Nathan Raw thanked the deputation and promised that the points suggested would have the attention of the Committee.

### Passing Events and Topics.

### Founders' Day at Alton.

To celebrate Founders' Day, the Lord Mayor will visit the Alton Cripples' Hospital on Saturday, June 4.

### Royal Hospital for Incurables.

Sir James Carmichael, K.B.E., will preside at a Festival Dinner in aid of the Royal Hospital and Home for Incurables, Putney, on Thursday, June 9. By the kindness of the Court of the Grocers' Company the dinner will be held at Grocers' Hall, Princes Street.

### Children's Hospital and the Music Hall Stage.

The hospitals of London owe much to the self-denying efforts of the music-hall and theatrical professions. Mr. Charles Coborn, the veteran music-hall artiste, who made himself famous by the well-remembered song, "The man who broke the bank at Monte Carlo," has come to the aid of the Belgrave Hospital for Sick Children, Clapham Road, and it is anticipated that as the result of his efforts a good sum has been raised by a concert promoted by him on behalf of its much-depleted funds.

### The Camera at St. George's Hospital.

The *Sphere* presents something of a novelty so far as British hospitals is concerned, by publishing in its issue of April 2 a series of photographs, occupying a double page, of the Treasurers, members of the medical, administrative, and nursing staffs, the Chaplain, the pharmacist, and the head porter of St. George's Hospital, together with a view of the elevation. The photographs are up to the *Sphere's* high standard, and there can be no doubt that they will prove of considerable interest to the public, to whom the workers in our great hospitals are little known.

### Points from Annual Reports.

### Hospital for Women.

Princess Christian has become President of the Hospital for Women, Soho, in the room of the Earl of Shaftesbury.

### The London Jewish Hospital.

The London Jewish Hospital have at present only an out-patient department, but it is well equipped and doing excellent work. Last year the average daily attendance was 120. The dental department receives many patients, and an almoner relieves those who need assistance, who were 5,859 in number last year.

### London Homœopathic Hospital.

The Duke of York, it was announced at the annual meeting, has consented to become patron of the London Homœopathic Hospital.

### The London Lock Hospital.

At the annual meeting of Governors of the London Lock Hospital and Rescue Home it was stated that in response to the appeal for £80,000 for the purpose of adding a new wing to the female hospital, to be devoted to maternity work, only £5,722 had been received. On maintenance account during 1920 there was a deficit of £417.

### Matrons' and Sisters' Appointments.

Beverley Dispensary and Hospital.—Miss Mabel E. Young has been appointed matron. She was trained at Hull Royal Infirmary, where she was later assistant matron.

General Hospital, Bridgwater.—Miss Durston has been appointed sister. She was trained at the Middlesex Hospital, and has taken sister's holiday duty there.

Royal Buckinghamshire Hospital, Aylesbury.—Miss D. J. Tudeney has been appointed sister of the women's and children's ward. She was trained at Guy's Hospital, and has the certificate of the Central Midwives Board. Miss Burgess has been appointed sister of the men's ward. She was trained at the Royal Berks Hospital, Reading, and has been sister at the Coventry and Warwickshire Hospital and at the Cornelia Hospital, Poole.

Children's Hospital, Birkenhead.—Miss Dorothy Tallis has been appointed ward sister. She was trained at the Royal Lancaster Infirmary and at Monsall Hospital, Manchester. She served under the British Red Cross in Roumania and Russia for five years, and has recently been staff nurse at Jessop Hospital, Sheffield.

Township of Leeds Infirmary.—Miss Mabel W. Gallant has been appointed maternity sister and teacher. She was trained at the Poplar and Stepney Sick Asylum, did military nursing from 1915 to 1919, has been ward and night sister at Kingston-on-Thames Infirmary, and more recently ward sister under the British Committee of the Russian Red Cross.

Royal Berkshire Hospital, Reading.—Miss Rose Thomas has been appointed home and night sister. She was trained at the Preston and County of Lancaster Royal Infirmary, where she was subsequently theatre sister; she has been night and day sister at the Royal Victoria Hospital, Bournemouth; day, night, and temporary home sister at Baschurch Orthopædic Hospital; and male ward and theatre sister at the Infirmary, Wrexham.

Chiswick and Ealing Isolation Hospital.—Miss Maud Alice Robinson has been appointed sister. She was trained at Bethnal Green Infirmary and Hastings Fever Hospital, at which latter she was subsequently sister. She has also served as sister under the British Red Cross Society.

Englethwaite Hall (T.B. Colony), near Carlisle.—Miss B. Hennessy has been appointed matron. She was trained at Spittals Hospital, Newcastle, Staffs, was sister T.F.N.S. from 1915 to 1919, health visitor under Cumberland County Council, and has recently been sister at Englethwaite Hall.

Wrenbury Hall Colony (Tuberculosis), Cheshire.—Miss Florence Keene has been appointed matron and Miss E. Robinson sister. Miss Keene was trained at the Derbyshire Royal Infirmary and at Monsall Hospital, Manchester; was sister and temporary assistant matron at the former institution, sister at Preston Borough Isolation Hospital, and has been matron of the Penistone District Isolation Hospital of the Winsley Sanatorium, near Bath, and of the Blencathra Sanatorium, Threlkeld, Cumberland. Miss Robinson was trained at the Royal Hants County Hospital, Winchester, where she was afterwards assistant matron and home sister. She has been sister at the Boston Hospital, Lincolnshire, temporary housekeeping sister at the Leicester Royal Infirmary, sister at Winsley Sanatorium, near Bath, and sister at Blencathra Sanatorium, Threlkeld. Both Miss Keene and Miss Robinson are members of the College of Nursing.

Putney Hospital.—Miss Dorothea Carey has been appointed sister. She was trained at Guy's Hospital and the

(*Continued on p.* vi.)

APR 27 1921 Surgeon General's Office

### The Hospital

**RATES OF SUBSCRIPTION** (post free, payable in advance).

Three months, 3/3; six months, 6/6; twelve months, 13/-. (Should the cost of printing and paper as well as increased postage, necessitate alteration of these rates, the period paid for will be reduced proportionately on unexpired subscriptions.) **THE HOSPITAL "Index" to Volume LXVIII., April to September 1920, is now ready, price 1/1 per copy, post free.** Address The Manager, The Hospital, "The Hospital" Building, 28/29 Southampton Street, Strand, London, W.C. 2.

### Uniform System of Accounts for Hospitals and Institutions

A complete and up-to-date Exposition of the Uniform System of Accounts for Institutions approved by King Edward VII. Hospital Fund, together with specimen rulings of the various books necessary to the scheme.

By Sir HENRY BURDETT, K.C.B., K.C.V.O.

Royal 8vo. Bound in cloth boards. **5/- net, by post 5/5.**

**THE SCIENTIFIC PRESS, LIMITED**
28/29 Southampton Street, Strand, London, W.C. 2.

